# Psychometrics of the modified Family-Centered Care Assessment short version for childhood obesity

**DOI:** 10.21203/rs.3.rs-4365570/v1

**Published:** 2024-05-15

**Authors:** Meg Simione, Paola Ferreira, Man Luo, Clarissa Hoover, Meghan Perkins, Lauren Fiechtner, Elsie M. Taveras

**Affiliations:** Mass General for Children; Mass General for Children; Mass General for Children; Family Voices; Mass General for Children; Mass General for Children; Mass General for Children

**Keywords:** Pediatrics, family-centered outcomes, patient experience

## Abstract

**Background:**

Incorporating principles of family-centered care into pediatric weight management interventions can improve the effectiveness and quality of treatment and reduce attrition rates. To assess the family-centeredness of interventions, reliable, valid, and easy-to-administer scales are needed. The purpose of the study was to develop a shortened version of the modifed Family Centered Care Assessment (mFCCA) and assess its psychometric properties.

**Methods:**

The mFCCA, a scale to assess the family-centeredness of interventions for childhood obesity, was administered to families following the Connect for Health randomized control trial evaluating the effectiveness of a primary care-based pediatric weight management intervention. We iteratively removed items from the mFCCA and used Rasch modeling to examine the reliability and validity of the shortened scale.

**Results:**

We included data from 318 parents and the exploratory factor analysis showed the presence of a single factor. The results of the Rasch modeling demonstrated acceptable internal consistency of the scale (0.7) and strong validity as evidenced by the overall model fit and range of item difficulty. Following the psychometric analyses, we reduced the number of items from 24 to 8 items.

**Conclusions:**

The mFCCA short version demonstrates good psychometrics and can be used to evaluate the family-centeredness of childhood obesity interventions with reduced participant burden, thereby improving outcomes for children with obesity.

**Trial registration:**

Clinicaltrials.gov NCT02124460 registered on April 24, 2014

## Background

The prevalence of obesity among children in the United States continues to be high (1, 2) and is higher among Hispanic and Black children compared to White and Asian children (3, 4). Similar disparities exist between low- and high-income groups (3, 4). Pediatric weight management interventions have become critical in preventing, managing, and reducing inequities in obesity treatment for children (5). However, retention rates for these interventions are low, with attrition rates ranging from 32 to 73%, thereby reducing their impact (6, 7). To improve the effectiveness and quality of interventions and reduce attrition rates, principles of family-centered care must be incorporated. Family-centered care is an approach that recognizes the important role that families play in their child’s health and aims to improve family and healthcare provider partnerships (8). It improves understanding and communication between families and providers, increasing parental program satisfaction, improving long-term health outcomes, and reducing attrition rates (9).

Reliable, valid, and easy-to-administer scales are needed to determine if an intervention is family-centered. The modified Family Centered Care Assessment (mFCCA) (10) is an adapted version of the Family Centered Care Assessment (FCCA) (11) tool for children with obesity that measures the family-centeredness of interventions. While the mFCCA has previously been found to be valid and reliable, it includes 24 items which is not practical for research purposes or clinical application (10). To overcome the administration burden, we sought to develop a shortened version that would promote greater uptake of the tool and the principles of family-centered care being incorporated into clinical practice, thereby reducing child obesity rates and improving health outcomes. This study aimed to develop a shortened version of the mFCCA and assess the tool’s psychometric properties.

## Methods

The data used to evaluate the psychometrics of the mFCCA short version was from the Connect for Health randomized control trial. The trial has previously been described in detail (12, 13). The one-year trial tested the effectiveness of two clinical-community interventions on improving body mass index (BMI) and quality of life. It enrolled 721 children ages 2–12 years with a BMI > = 85th percentile and was conducted in pediatric primary care practices in Massachusetts. The enhanced primary care arm (n = 361) included clinical decision support tools to alert clinicians to elevated BMIs and guide best practice management, family educational materials, neighborhood resource guides, and a social- and community-informed text messaging program. The health coaching arm (n = 360) received the same enhancements as the other arm in addition to contextually tailored health coaching support. Both arms were found to effectively reduce BMI and improve quality of life (13). Parents of children in the enhanced primary care arm answered questions relating to their primary care provider, and therefore, only data from that arm were included in the psychometric analysis as it is more typical of primary care. The Mass General Brigham institutional review board approved the trial.

### Development of the Shortened FCCA

The mFCCA was adapted from the FCCA, and the adaptation process and psychometric analysis have previously been described (10, 11). Briefly, the mFCCA has 24 items representative of principles of family-centered care and includes questions from eight topical areas (communication, future promotion, decision-making, strength-based, practice structure, family support, care coordination, and cultural competence). Ordinal responses range from 1 to 5, with higher scores indicating a greater perception of family-centeredness, as well as a “not applicable” response.

The length of the scale remained a barrier to uptake for use in research and clinical practice, and therefore, we aimed to shorten it. Three experts in childhood obesity reviewed the items and selected nine items. Items selected represented a range of topical areas and item difficulty. The process was done in consultation with a member of the original FCCA creator.

### Psychometric Analyses

Following the selection of the nine items, we used Rasch modeling to examine the psychometrics of the shortened scale (14, 15). The original version of the FCCA and the mFCCA used item response theory to examine the reliability and validity of the tool (10, 11). We began by performing an exploratory factor analysis using the principal axis method on the nine items to confirm the unidimensionality of the scale.

We determined that any item with a factor loading < 0.4 would be deleted (11). Using a Scree plot, we reviewed the eigenvalues indicating the number of factors. To measure reliability and the homogeneity of the scale, we calculated the item total correlations and deleted correlations < 0.3. We then used a partial credit model to assess the overall fit of the items and calculated item fit statistics to examine how well the data fit the model (16–18). We determined infit and outfit statistics to detect inliers and outliers, and the criteria range was 0.5–1.5 (19, 20). Standard error and item difficulty were calculated for each item. Item difficulty is represented on a logit scale and ranges from negative, which represents easy items that could easily be incorporated into care, to positive, which represents difficult items that would be more challenging to incorporate into care (14). We examined potential question bias by performing the Differential Item Functioning (DIF) to understand if an item measures different abilities for subgroups (sex, income, race, and ethnicity). We would expect pseudo R^2^ measures to be < 0.02 in a no DIF condition (21, 22). To assess the scale’s internal consistency, we calculated a person separation reliability (equivalent to a Cronbach’s alpha) (23). After conducting the analyses, we reviewed the results to ensure they fell within the predetermined acceptable ranges. All items fell within acceptable ranges therefore none were removed based on those criteria. We then removed one additional item (reduced to eight items) to attempt to shorten the scale further and selected an item that would not affect the range of item difficulty. We repeated the analyses, and again, all items fell within acceptable ranges. We repeated this procedure for an additional item (reduced to seven items) and found that the person separation reliability decreased, therefore we opted to keep the scale at eight items to retain its strong psychometric properties. We then calculated a score by averaging responses for the final eight items as was done in the mFCCA. R version 3.4.4 and the eRM and lordif packages were used to perform analyses (16, 21, 24)

## Results

We included 318 parents in the analyses from the enhanced primary care arm. The trial had 721 participants, and 638 parents completed the mFCCA, of which 323 participants were in the enhanced primary care arm. Five parents were excluded from the analyses as > 50% of items were missing or “not applicable”. Table 1 shows the child and parent characteristics. The mean (SD) age of children was 8.0 (3.0) years with a mean (SD) BMI of 22.9 (4.6). The race and/ or ethnicity of children were 38% White, 31% Black or African American, and 22% Hispanic or Latino, and 38% of children’s households had an income ≤ $50,000.

### Psychometric Analyses

**Figure 1:**
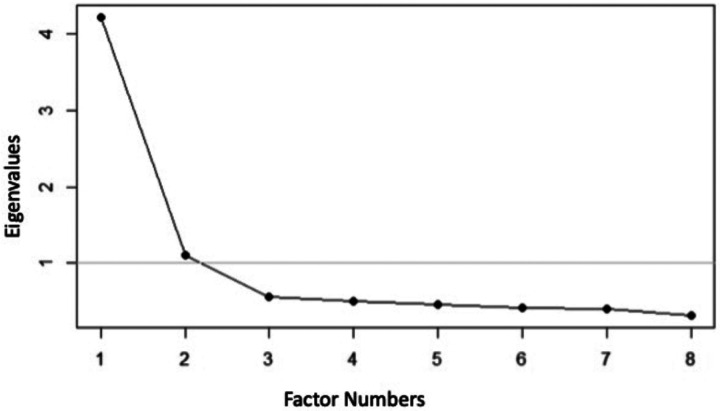
Exploratory factor analysis Scree plot of the modified Family-Centered Care Assessment Tool short version.

The results of the psychometric analyses are shown in Table 2. The exploratory factor analysis showed the presence of a single factor (eigen value = 3.7) explaining 46% of the variance (see Fig. 1). All individual item factor loadings were > 0.4, and all item total correlations for individual items were > 0.3. The eight items fell between the range of 0.5–1.5 for the Rasch item fit statistics. Item difficulty revealed a broad range from − 1.2 logits (representing the easiest questions) to 0.9 logits (representing the most difficult questions). We did not find bias for sex, income, race, and ethnicity when completing the DIF analyses as the pseudo R^2^ measures were < 0.02. In the final step, we found acceptable internal consistency of the scale (0.7) when calculating the person separation reliability. The mean (SD) score was 3.81 (1.04). The final version of mFCCA is shown in **Supplemental Table 1**.

## Discussion

In this study, we shortened the mFCCA from 24 to 8 items and found the 8-item version to have good reliability and validity. A shortened scale allows for quick administration by research and clinical programs to assess the family-centeredness of interventions. Evaluation tools with strong psychometrics can promote family-centered care and ultimately improve participation and outcomes for children with obesity.

The psychometrics of the mFCCA short version were good; we found similarities and differences between this version and the 24-item mFCCA (10). Both scales resulted in similar mean scores as the mFCCA had a mean (SD) score of 3.84 (0.95), and the short version had a score of 3.81 (1.04). The two most notable differences were the item difficulty and person separation reliability. Item difficulty requires a range of questions that would be easy to incorporate into care to questions of increasing difficulty. Although we had a range of items from easy to difficult, compared to the mFCCA, the range was condensed with the difficult questions decreasing from 1.10 to 0.92. A range of questions with differing item difficulty allows the scale to discern differences between interventions with high and low family-centered practices (14). We also found the person separation reliability to change, moving from high to acceptable internal consistency of the scale.

When reducing the number of items in a scale, we recognize that there will be a trade-off (25). We had to balance having a scale with good psychometrics that was also easy to administer as 24 items are not feasible for researchers and clinicians evaluating interventions. Finding this balance was important, as to our knowledge, there are no other scales that assess family-centeredness for childhood obesity interventions in primary care, and to promote family-centered care we require tools to evaluate it (26).

Family-centered care has shown improved outcomes in childhood obesity interventions, such as BMI reduction, health behaviors, and quality of life (27), and decreased attrition (6, 9). Family-centered care has also been found to improve family members’ well-being as well as healthcare providers’ satisfaction (28). Additionally, it can improve health equity by empowering parents to discuss and address social determinants of health (29). Given the racial, ethnic, and socioeconomic disparities that persist in the rates of obesity (3, 4), methods to eliminate disparities are vital, and ensuring interventions are family-centered can help address health disparities. When selecting items for the shortened version, we were cognizant of the systemic reasons for obesity (30) and purposefully selected items that focused on ways to address those barriers (for example, “has a way to help me contact community resources”).

Our study is not without limitations. Our sample is from one healthcare system in the Greater Boston area, which may not be representative of the United States. The participants were also participating in a randomized controlled trial, which, again, may not reflect all families who attend primary care. Additionally, this is the same sample that was used when assessing the psychometrics for the mFCCA. Future studies should continue to assess the psychometrics in other populations.

## Conclusions

The mFCCA short version demonstrates good psychometrics and can be used to evaluate the family-centeredness of childhood obesity interventions. Administering an eight-item scale is feasible for both researchers and clinicians, and by evaluating interventions, we can encourage family-centered care and thereby improve health outcomes for children with obesity.

## Figures and Tables

**Table 1:** Child, parent, and household characteristics of the usual care intervention arm that were included in the psychometric analyses (n = 318)

Child Characteristics	No. (%)
Age (years), mean (SD)	8.0 (3.0)
Sex	
Male	148 (46.5)
Female	170 (53.5)
Race and/ or ethnicity	
White	120 (37.7)
Black or African American	100 (31.4)
Hispanic or Latino	71 (22.3)
Other^a^	27 (8.49)
BMI, mean (SD)	22.9 (4.6)
BMI z-score, mean (SD)	1.91 (0.5)
Parent Characteristics	No. (%)
Age (years), mean (SD)	38.8 (7.6)
BMI	
<25	71 (23.7)
25–29	211 (70.3)
≥ 30	18 (6.0)
Income	
≤ 50k	118 (37.7)
>50k	196 (62.3)
Education	
Some college or less	163 (51.3)
College graduate or more	155 (48.7)

a American Indian or Alaska Native, Asian, Native Hawaiian or Pacific Islander

**Table 2:** Estimates of item difficulty, standard error, mean-square fit statistics, item–total correlations, and topical area of the Modified Family-Centered Care Assessment short version

mFCCA Item #	My child’s health care provider/health coach…	Item Difficulty	Standard Error of Item Score	Infit Mean-Square Value	Outfit Mean-Square Value	Item Total Correlation	Topical Area
4	Takes enough time to address my concerns	−1.23	0.03	0.91	1.19	0.53	Communication
12	Recognizes my strengths in caring for my child	−0.85	0.04	0.85	0.83	0.60	Strength-Based
3	Decides together on goals	−0.45	0.05	0.88	0.93	0.62	Decision Making
11	Asks me what is working well	−0.51	0.04	0.63	0.54	0.71	Strength-Based
14	Asks me about health or emotional stresses I have	−0.04	0.06	0.93	0.91	0.64	Family Support
19	Has a way to help me contact community resources	0.06	0.06	0.84	0.78	0.69	Care Coordination
17	Asks about my family’s beliefs and practices	0.45	0.07	0.97	1.12	0.60	Cultural Competence
20	Has a way to connect me with other families	0.92	0.08	1.03	1.00	0.54	Family Support

Note. mFCCA = Modified Family-Centered Care Assessment Tool

## Data Availability

The datasets used and/or analyzed during the current study are available from the corresponding author on reasonable request.

## Abbreviations

BMI: Body mass index
DIF: Differential item functioning
FCCA: Family Centered Care Assessment
mFCCA: Modified Family Centered Care Assessment
